# Supplementary material for: Stool Serology: Development of a Non-Invasive Immunological Method for the Detection of Enterovirus-Specific Antibodies in Congo Gorilla Faeces
Source: Microorganisms. 2021 Apr 12;9(4):810. doi: 10.3390/microorganisms9040810 (PMC8068960; doi:10.3390/microorganisms9040810)
Supplement: Supplementary file 1 [file microorganisms-09-00810-s001.pdf]

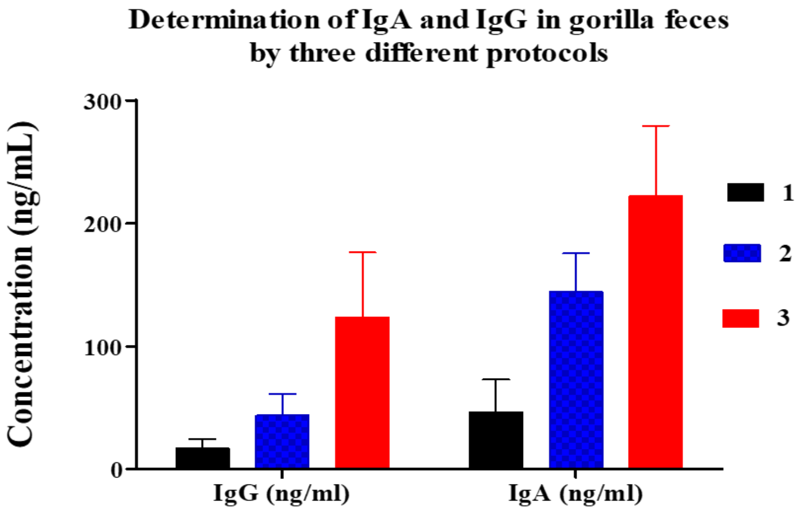

Figure S1. Quantification of immunoglobulins (IgG and IgA) in gorilla faeces with three different faecal treatment protocols. (1) Filtration of stool extract from 2 g of stools plus 2 ml of buffer; (2) Lyophilisation of the filtered extract, reconstituted in 500 µl of buffer and then purification of IgG and IgA in the reconstituted extract with protein G and pectin M; (3) Lyophilisation of the filtered extract, reconstituted in a small volume of buffer (500 µl) then concentrated by the Amicon® Ultra-1.
